# MicroRNA Alterations for Diagnosis, Prognosis, and Treatment of Osteoporosis: A Comprehensive Review and Computational Functional Survey

**DOI:** 10.3389/fgene.2020.00181

**Published:** 2020-03-03

**Authors:** Hai Hu, Xiaodi He, Yazhong Zhang, Rongrong Wu, Jiajia Chen, Yuxin Lin, Bairong Shen

**Affiliations:** ^1^Center for Systems Biology, Soochow University, Suzhou, China; ^2^Department of Orthopedics, Huainan First People’s Hospital of Anhui Province, Huainan, China; ^3^School of Medicine, Anhui University of Science and Technology, Huainan, China; ^4^Department of Orthopedics, The First Affiliated Hospital of Soochow University, Suzhou, China; ^5^School of Chemistry, Biology and Material Engineering, Suzhou University of Science and Technology, Suzhou, China; ^6^Institutes for Systems Genetics, West China Hospital, Sichuan University, Chengdu, China

**Keywords:** osteoporosis, microRNA alteration, integrated miRNA-gene-pathway analysis, systems biology, precision medicine

## Abstract

Osteoporosis (OP) is a systemic bone disease with a series of clinical symptoms. The use of screening biomarkers in OP management is therefore of clinical significance, especially in the era of precision medicine and intelligent healthcare. MicroRNAs (miRNAs) are small, non-coding RNAs with the potential to regulate gene expression at the post-transcriptional level. Accumulating evidence indicates that miRNAs may serve as biomarkers for OP prediction and prevention. However, few studies have emphasized the role of miRNAs in systems-level pathogenesis during OP development. In this article, literature-reported OP miRNAs were manually collected and analyzed based on a systems biology paradigm. Functional enrichment studies were performed to decode the underlying mechanisms of miRNAs in OP etiology and therapeutics in three-dimensional space, i.e., integrated miRNA–gene–pathway analysis. In particular, interactions between miRNAs and three well-known OP pathways, i.e., estrogen–endocrine, WNT/β-catenin signaling, and RANKL/RANK/OPG, were systematically investigated, and the effects of non-genetic factors on personalized OP prevention and therapy were discussed. This article is a comprehensive review of OP miRNAs, and bridges the gap between an understanding of OP pathogenesis and clinical translation.

## Introduction

According to the World Health Organization definition, OP is a systemic bone disease with the characteristics of decreased bone mass, bone destruction, decreased bone mineral density (BMD), increased bone fragility, and a high risk of fracture that may be caused by mild trauma or even in the absence of trauma (Ivanova et al., 2015). OP is well-known as a silent disease without obvious clinical symptoms before a bone is found to be fractured following minor trauma, and has become one of the most important public health problems in the world.

miRNAs widely exist in eukaryotes. They are a class of small non-coding RNAs with about 21 to 24 nucleotides, and hold the power to regulate gene expression at the post-transcriptional level. Accumulating evidence has convinced that miRNAs are functional players in multiple cellular abilities and biological processes, such as the proliferation, cycle, and apoptosis of cells; the development of complex diseases, including cancers, cardiovascular diseases, and neurodevelopmental diseases, is often closely related to the deregulation of miRNAs. In recent years, extensive efforts have been made to show that miRNAs play important roles in OP occurrence and development, especially in the osteoporotic fracture. For instance, miRNAs are involved in bone metabolism in OP through regulating target genes associated with human mesenchymal stem cells (hMSCs), bone cells osteoblasts and osteoclasts, which are important components during bone remodeling (Feng et al., 2018). Reported miR-19a-3p promotes osteogenic differentiation of hMSCs by inhibiting HDAC4 expression, thereby reducing the progression of OP (Chen et al., 2019). Seeliger et al. (2014) found that the expression of RUNX2 in OP could be co-regulated by miR-23a, miR-24, and miR-27a, thereby contributing to osteoblast dysfunction. Krzeszinski et al. (2014) indicated that miR-34a was an inhibitor suppressing osteoclast differentiation by directly targeting transforming growth factor-β-induced factor 2 (Tgif2) and its associated osteoclastogenesis.

Biomarkers or markers are measurable and evaluable substances that can indicate change within biological systems. They are traceable and can predict disease occurrence and progression with high sensitivity and specificity, therefore exhibiting great potential for the diagnosis, prognosis, and therapy of various human diseases, including OP. With advances in biomedical research, plenty of studies have reported miRNAs as biomarkers for OP management, and reviews on OP miRNA biomarkers have been conducted to further the understanding of OP pathogenesis. For example, Cheng et al. (2019) summarized the characteristics of miRNA in bone metabolism, the progress of OP, and the prospect of clinical treatment, which provided significance for OP screening, monitoring, and prognosis. In addition, a miRNA-based tool for risk assessment of OP fracture can reduce the incidence of fracture (Walter et al., 2018). As a reliable bone biomarker of OP, miRNAs provides a new idea for the treatment of OP and helps to reduce the risk of fracture (Garnero, 2017). However, most of these studies have focused solely on the expression level of miRNAs in OP samples (tissue, blood, and cell line) with few providing deeper insight into the regulatory patterns of miRNAs, i.e., from miRNAs, target genes, to integrated pathways. OP is complex and heterogeneous in nature; thus decoding its development using a systems biology framework, to enable personalized OP diagnosis and treatment, is of great significance.

This study provides a comprehensive review of current frontiers in the use of miRNA alterations for precision OP medicine and management. First, the definition of OP and its clinical symptoms are briefly introduced and its underlying pathogenic mechanisms presented. In particular, three crucial pathways, i.e., the estrogen–endocrine pathway, the WNT/β-catenin signaling pathway, and the receptor activator of nuclear factor-κB ligand (RANKL)/receptor activator of nuclear factor-κB (RANK)/osteoprotegerin (OPG) pathway, are emphasized as clues for systems-level “miRNA–gene–pathway–OP” deciphering. Second, reported miRNA alterations are manually collected and summarized by integrating OP sample source information (e.g., tissue, blood, and cell line) and subtype classifications [e.g., post-menopausal OP (PMO), senile OP (SOP)]. The targets of the reported OP miRNAs are computationally identified and investigation into OP pathogenesis is performed at gene, pathway, and cross level (i.e., integrating genetic and non-genetic factors for OP understanding). Here the relationship between the reported miRNA dysregulation and the three OP-associated pathways is explored, based on a combination of functional enrichment analysis and literature survey. Finally, challenges and perspectives for the integration and fusion of OP data and knowledge for future clinical translation and personalized OP healthcare are discussed.

### Disease and Symptoms

**FIGURE 1:**
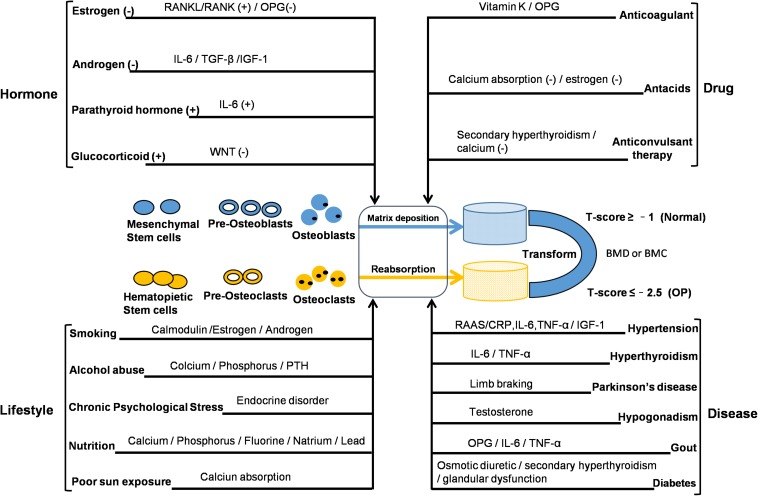
Interactions between genetic and non-genetic factors in OP development. The reported factors may change the balance of bone remodeling. Bone density and structural integrity are dependent on bone-remodeling mechanisms associated with the function of osteocytes, osteoblasts, and osteoclasts. BMC, bone mineral content; BMD, bone mineral density; CRP, C-reactive protein; IL, interleukin; IGF, insulin-like growth factor; OPG, osteoprotegerin; PTH, parathyroid hormone; RAAS, renin angiotensin aldosterone system; RANK, receptor activator for nuclear factor-κB; RANKL, receptor activator for nuclear factor-κB ligand; TGF, transforming growth factor; TNF, tumor necrosis factor; (+): increase or promote; (–): reduce or inhibit.

Osteoporosis is a systemic bone disease characterized by decreased BMD or bone mineral content, resulting in increased bone fragility and an increased tendency to fracture. The quality and density of bone are constantly improved by the process of bone remodeling. Bone remodeling is bone formation by osteoblasts and the resorption of osteoclasts. It is a process of repeated renewal that eventually leads to a stable skeletal state. According to a recent epidemiological survey, about 200 million people worldwide have OP, and about 9 million of them have osteoporotic fractures. The risk of fracture in OP patients is up to 40%, and spine, hip, and wrist are especially prone to osteoporotic fractures. The mortality of osteoporotic patients is high due to fractures of the spine and hip. OP is a chronic disease with many causative factors. According to the clinical classification, OP can be divided into two types: primary and secondary. Primary OP can be classified into consists of three subtypes – that type I PMO, type II SOP, and type III idiopathic OP, with fracture the most common complication. Secondary OP is OP arising as a complication of a primary condition. Prior to fracture, there is usually no clinical manifestation. The disease is more common in women than in men, and is most common in post-menopausal women and the elderly. Interactions between genetic and environmental factors play an important role in its occurrence and development (see Figure 1). The pathogenesis of OP can be summarized into two aspects. The first is the growth stage, during which higher peaks of BMD are not reached, resulting in poor bone strength. The second aspect is bone formation, with bone resorption caused by bone loss and an imbalance caused by bone structure damage. The pathophysiology of OP mainly relates to bone cells – osteoblasts and osteoclasts. The reason for the occurrence of OP is not completely clear; however, several studies have indicated that its pathogenesis is closely related to a number of factors. These include (1) regulation by hormones; (2) lifestyle factors; (3) disease; (4) drugs. In addition, OP is influenced by genetic factors and patients with severe OP often have a family history. The mechanism of bone loss in OP caused by cancers should be noticed. The reasons include the bone metastasis of cancer invading bone microenvironment, leading to the imbalance of bone homeostasis or the change of bone structure, as well as the bone destruction resulted from the treatment of cancer (Coleman et al., 2014). Recine et al. (2019) showed that the management of bone health in breast cancer patients after receiving endocrine therapy is necessary and the decrease of bone mass or OP caused by the imbalance of bone homeostasis may be related to estrogen deficiency and lifestyle. In addition, antiandrogen therapy for patients with prostate cancer increased the risk of osteoporotic fracture (Wallander et al., 2019).

Recent studies on OP have focused on the estrogen, RANKL/RANK/OPG (OPG: osteoprotegerin), and WNT signaling pathways. These three signaling pathways have their own signal transduction targets and are closely interrelated, forming a complex system to regulate bone metabolism in OP. Earlier observations showed that estrogen has a therapeutic effect on PMO, with the bone mineral content of 63 ovariectomized patients in one study increasing significantly following estrogen treatment (Lindsay et al., 1976). The protective effect of estrogen on bone is mainly mediated by the estrogen signaling pathway, which promotes the proliferation and activity of osteoblasts and inhibits the activity of osteoclasts. The estrogen/estrogen receptor α (ER-α) signal plays a positive role in the metabolism of osteoblasts. Estrogen has been shown to have a direct inhibitory effect on the bone resorption of osteoclasts. Additionally, through experimental comparison of ovariectomized mice and sham-operated mice, it is evident that estrogen indirectly regulates the production of bone marrow stromal cells (BMSCs), through macrophage colony-stimulating factor (MCSF) induced by interleukin 1 (IL-1) and tumor necrosis factor (TNF), and that BMSCs play a key role in the proliferation and differentiation of osteoclast precursors (Kimble et al., 1996), which also involves osteoclasts induced by the RANKL/RANK/OPG network pathway. The role of the RANKL/RANK/OPG signaling pathway network in regulating bone metabolism was discovered at the end of the last century. Simonet et al. (1997) initially discovered that osteoprotegerin (OPG: the bone protector) was associated with osteosclerosis in mice, blocking osteoclast formation and affecting bone resorption. The reason is that OPG can inhibit bone resorption of osteoclasts by the RANKL/RANK signaling pathway. In addition, the ratio of OPG to RANK is related to bone mass (Boyce and Xing, 2007). RANK is an essential component of osteoclast production mediated by transmembrane proteins, and this process requires activation of extracellular RANKL binding. The cytokine RANKL expressed in osteoblasts plays an important role in the bone resorption of osteoclasts, which involves osteoclast differentiation and activity mediated by the RANKL/RANK signaling pathway, a key link in the process of bone reconstruction (Ikeda et al., 2001). In particular, OPG can bind to RANKL, thereby reducing the positive effect of RANK/LRANK on osteoclasts (Boyce and Xing, 2007). The WNT signal affects changes in the structure of the tissue by participating in various aspects of the cell life process (Laine et al., 2013). Through a controlled study of a family, Keupp et al. (2013) found that dominant mutation of WNT1 was related to early onset OP. The WNT signaling pathway plays an important role in bone remodeling and bone mass stabilization. In particular, WNT-regulated β-catenin signaling stimulates osteoblast differentiation and increases bone mass, and in the β-catenin signaling transduction pathway. WNT activates the LRP5/6 and Frizzled receptor complexes on the cell membrane and polymerizes under the action of Axin and APC proteins. The receptor complexes inactivate the phosphorylation of glycogen synthase and prevent the degradation of β-catenin. Deactivation ensures that β-catenin enters the nucleus and acts together with TCF/LEF to mediate the transcriptional expression of related genes and promote the positive regulation of osteoblasts. Dickkopf-related protein 1 (DKK1) acts as an inhibitor of the WNT signaling pathway. DKK1 mediates rapid endocytosis by combining with Kremen2 and LRP5/6 to form a trimer, which reduces the amount of LRP5/6 on the cell membrane and prevents the WNT signaling pathway from transmitting to the cell (Asally and Yoneda, 2005; Patel et al., 2018). The role of glycogen synthase kinase 3β (GSK-3β) in the cytoplasm is phosphorylating β-catenin and promoting its degradation by ubiquitin proteolytic system. The β-catenin in the nucleus, acting on T-cell specific transcription factor (TCF) and lymphoid enhancer binding factor 1 (LEF-1) to regulate transcription of related target genes (Behrens et al., 1996). Clinical dual energy X-ray bone densitometry (DXA) is the gold standard for determining OP. In general, BMD or bone mineral content is within a standard deviation of the average *t*-value of normal adults. OP is defined as BMD less than the normal value of 2.5 standard differences; if accompanied by a brittle fracture, it is considered serious OP. The diagnosis of PMO and SOP should be differentiated from osteomalacia, myeloma, osteogenesis imperfecta, and various cancerous osteopathy. The treatment of OP in western medicine is by basic supplementation and drug treatment. Supplementation mainly involves calcium and vitamin D, with a recommended dose for adults of 800 mg and 200 IU daily, respectively. For the elderly, the recommended daily intake is increased to 1000 mg and 400–800 IU, appropriately adjusted according to dietary intake and regular monitoring of calcium and urine calcium. For Chinese elderly people, according to the average dietary intake, calcium should be supplemented by 500–600 mg daily. Simultaneously, appropriate physical exercise and the avoidance of smoking are also recommended. The reduction of alcohol intake is also important. Drug therapy includes drugs that prevent bone resorption and promote bone formation. Anti-bone resorption drugs include (1) bisphosphonates, which can effectively inhibit osteoclast activity – clinically, alendronate, for example in the form of alendronate sodium, can be taken orally in the morning, although it can result in side effects affecting the digestive tract; (2) calcitonin, which effectively inhibits the activity of osteoclasts and reduces their number – clinically, salmon and eel calcitonin are used; (3) selective ER modulators (SERMs), which effectively inhibit osteoclast activity and reduce bone resorption, an example being raloxifene are used in the prevention and treatment of PMO; (4) estrogens, which can inhibit bone resorption and bone transformation. Drugs that promote bone formation include parathyroid hormone (PTH): a low dose of PTH can increase the number of bone cells, thereby increasing BMD. For the vast majority of patients, treatment only begins after they sustain a severe osteoporotic fracture; general drug and surgical treatment is only symptomatic, managing short-term pain and fractures. Controlling the development of OP is more difficult; thus, making early diagnosis and the prevention and specific treatment of OP is particularly important and necessary.

### MiRNA Alteration for OP Monitoring

#### Search and Filtering Criteria

**FIGURE 2:**
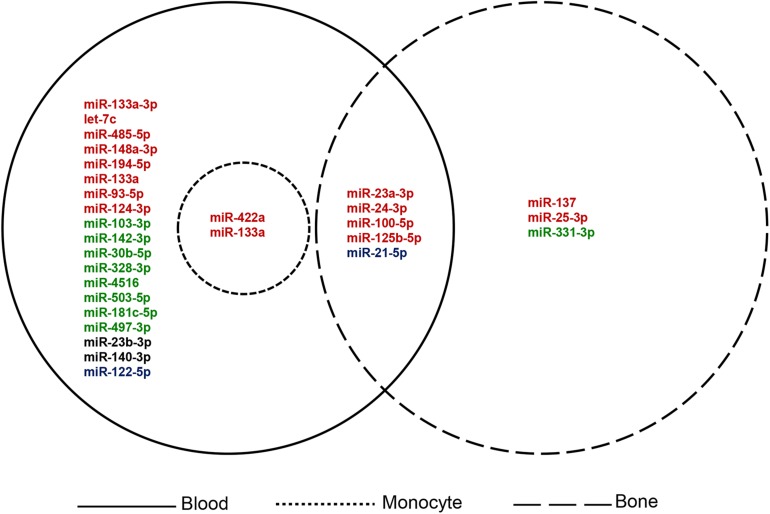
Classification of the collected OP miRNAs based on sample source. Red: up-regulated; green: down-regulated; blue: up-regulated and down-regulated in different studies; black: expression level not available.

The search terms used for the collection of information on previously reported OP miRNAs were “osteoporosis[tiab] AND (miRNA^∗^[tiab] OR microRNA^∗^[tiab]) AND (biomarker^∗^[tiab] OR marker^∗^[tiab] OR indicator^∗^[tiab] OR predict^∗^[tiab] OR therapeutic target^∗^[tiab]).” As shown in Table 1, a total of 41 items comprising 28 miRNAs were collected as key players for OP diagnosis, prognosis, or treatment (based on the summary and conclusion of the article). Those miRNAs reported as biomarkers or candidate/potential/latent biomarkers were considered, including experimental samples derived from blood, bone tissue, and special cell lines. Classification of the collected miRNAs based on different sample sources is illustrated in Figure 2.

**TABLE 1:** Literature-reported osteoporosis miRNAs.

Report ID	Official symbol	Osteoporosis type	Expression	Sample	Experimental method	AUC	PMID
miR-181c-5p	miR-181c-5p	PMO	Down	Blood	RT-PCR	NA	31872255
miR-497-3p	miR-497-3p	PMO	Down	Blood	RT-PCR	NA	31872255
miR-133a-3p	miR-133a-3p	PMO	Up	Blood	NA	NA	31023966
let-7c	let-7c	PMO	Up	Blood	NA	NA	30379578
miR-23b-3p	miR-23b-3p	PMO	NA	Blood	qRT-PCR	NA	30171938
miR-140-3p	miR-140-3p	PMO	NA	Blood	qRT-PCR	NA	30171938
miR-485-5p	miR-485-5p	NA	Up	Blood	qRT-PCR	NA	30070309
miR-122-5p	miR-122-5p	NA	Down	Blood	RT-qPCR	0.666	29849050
hsa-miR-4516	miR-4516	NA	Down	Blood	RT-qPCR	0.727	29849050
miR-148a-3p	miR-148a-3p	NA	Up	Blood	qPCR	NA	27900532
miR-30b-5p	miR-30b-5p	PMO	Down	Blood	qPCR	0.793	27821865
miR-103-3p	miR-103-3p	NA	Down	Blood	qPCR	0.8	27821865
miR-142-3p	miR-142-3p	NA	Down	Blood	qPCR	0.789	27821865
miR-328-3p	miR-328-3p	NA	Down	Blood	qPCR	0.874	27821865
miR-122-5p	miR-122-5p	NA	Up	Blood	RT-PCR	NA	26163235
miR-125b-5p	miR-125b-5p	NA	Up	Blood	RT-PCR	NA	26163235
miR-21-5p	miR-21-5p	NA	Up	Blood	RT-PCR	NA	26163235
miR-194-5p	miR-194-5p	PMO	Up	Blood	qRT-PCR	NA	26038726
miR-21	miR-21-5p	PMO	Down	Blood	NA	NA	25231354
miR-133a	miR-133a	PMO	Up	Blood	NA	NA	25231354
miR-21	miR-21-5p	NA	Up	Blood	qPCR	0.63	24431276
miR-23a	miR-23a-3p	NA	Up	Blood	qPCR	0.63	24431276
miR-24	miR-24-3p	NA	Up	Blood	qPCR	0.63	24431276
miR-93	miR-93-5p	NA	Up	Blood	qPCR	0.68	24431276
miR-100	miR-100-5p	NA	Up	Blood	qPCR	0.69	24431276
miR-122a	miR-122-5p	NA	Up	Blood	qPCR	0.77	24431276
miR-124a	miR-124-3p	NA	Up	Blood	qPCR	0.69	24431276
miR-125b	miR-125b-5p	NA	Up	Blood	qPCR	0.76	24431276
miR- 148a	miR-148a-3p	NA	Up	Blood	qPCR	0.61	24431276
miR-503	miR-503-5p	PMO	Down	Blood	qRT-PCR	NA	23821519
miR-137	miR-137	NA	Up	Bone	RT-PCR	NA	29786747
miR-331	miR-331-3p	NA	Down	Bone	NA	NA	26329309
miR-21	miR-21-5p	NA	Up	Bone	qPCR	0.63	24431276
miR-23a	miR-23a-3p	NA	Up	Bone	qPCR	0.63	24431276
miR-24	miR-24-3p	NA	Up	Bone	qPCR	0.63	24431276
miR-25	miR-25-3p	NA	Up	Bone	qPCR	NA	24431276
miR-100	miR-100-5p	NA	Up	Blood	RT-PCR	0.89	31532098
miR-100	miR-100-5p	NA	Up	Bone	qPCR	0.69	24431276
miR-125b	miR-125b-5p	NA	Up	Bone	qPCR	0.76	24431276
miR-422a	miR-422a	PMO	Up	Monocyte	qRT-PCR	NA	24820117
miR-133a	miR-133a	PMO	Up	Monocyte	qRT-PCR	NA	22506038

*AUC, area under receiver operating characteristic curve; NA, not available; PMO, post-menopausal osteoporosis; qRT-PCR, quantitative reverse transcription polymerase chain reaction; RT-qPCR, real-time quantitative polymerase chain reaction.*

The filtering criteria for miRNA collection is: (1) Sample sources must originate from human; (2) Circulating miRNA expression profiles of OP patients should be used; (3) Samples should be separated into case and control groups; (4) The expression of miRNA should mainly be validated through low-throughput (e.g., qRT-PCR) methods.

#### Overview of the Collected miRNAs

Through literature searching, we identified several miRNAs for OP diagnosis. Among them, ROC curves were performed for some prominently regulated miRNAs. The area under the curve (AUC) is used to determine the diagnostic value of each miRNA. The AUC values were higher than 0.6, which indicated their diagnostic value for OP. For example, Chen et al. (2016) found that miR-30b-5p, miR-103-3p, miR-142-3p, and miR-328-3p were down-regulated in PMO through animal experiments and analysis of serum miRNA validation in OP patients. By contrast, several miRNAs, i.e., miR-21 (also known as: miR-21-5p), miR-23a (also known as: miR-23-3p), miR-24 (also known as: miR-24-3p), miR-93 (also known as: miR-93-5p), miR-122-5p, miR-124a (also known as: miR-124-3p), miR-125b (also known as: miR-125b-5p), miR-100 (also known as: miR-100-5p) and miR-148a (also known as: miR-148a-3p), were up-regulated in serum samples of osteoporotic patients. Meanwhile, Seeliger et al. (2014) demonstrated that miR-21, miR-23a, miR-24, miR-25, miR-100 and miR-125b were highly expressed in OP bone tissue. Mandourah et al. (2018) validated the up-regulation of miR-122-5p and miR-4516 in OP patients by RT-qPCR based on five subgroups of human blood sample control studies, i.e., non-OP controls, osteopenia without fracture, osteopenia with fracture, OP without fracture, OP without fracture. In another mate-analysis, samples collected from 327 PMO patients and 328 post-menopausal health samples were analyzed, and robust rank aggregation (RRA) was used to determine the up-regulation of miR-133a-3p in the blood of OP patients (Pala and Denkceken, 2019). Ma et al. (2019) verified that miR-181c-5p and miR-497-3p were down regulated in the blood of 86 post-menopausal women with OP or osteopenia, and increased after treatment with bisphosphonate and calcitriol, suggesting the role of diagnosis and treatment of OP.

There are some miRNAs that are significant for the potential treatment of OP. For example, miR-503 (also known as: miR-503-5p) in circulatory mononuclear cells played an important role in the regulation of bone resorption in PMO. The overexpression of miR-503 inhibits osteoclast formation induced by RANKL, making it a potential target for OP treatment (Chen C. et al., 2014). Zhou et al. (2019) found that let-7c was up-regulated in the serum of PMO by targeting stearoyl-CoA desaturase 1 (SCD-1), which directly regulated the osteogenic differentiation of mesenchymal stem cells (MSCs) and provided a new potential therapeutic target for PMO. Zhang et al. (2018) found the up-regulation of miR-485-5p in OP patients in clinical case-control and *in vitro* cell experiments. They confirmed that Osterix was a direct target of this miRNA to regulate the expression of osteogenic markers by Luciferase assays, which indicated the potential therapeutic effect on OP.

We found that miR-137 could serve as a prognostic biomarker for OP. This miRNAs were derived from cells in bone tissue samples of OP patients. Liu and Xu found that the expression of miR-137 in osteoporotic fracture patients was significantly higher than that without fracture (Liu and Xu, 2018). In addition, RT-PCR and immunofluorescence experiments confirmed that the over-expression of miR-137 was associated with the occurrence of osteoporotic fracture, which was a potential biomarker for prognosis of the risk osteoporotic fracture (Liu and Xu, 2018).

Some miRNAs have also been proved to be potential biomarkers of OP. For example, Wang et al. (2012) and Cao et al. (2014) using qRT-PCR examined the expression of miRNAs in 10 post-menopausal women with high BMD and 10 controls women with low BMD, and found circulating monocytes of miR-133a and miR-422a were upregulated in low BMD. The target genes, including CXCL11, CXCR3, SLC39A1 and CBL, CD226, IGF1, PAG1, TOB2, related to osteoclasts and the negative expression of miR-133a and miR-422a were predicted and verified by up-regulation in cells. Li et al. (2014) studied the expression of plasma miRNA in 120 post-menopausal women (divided into three groups: normal, osteopenia, OP) and found that miR-21 and miR-133a increased significantly in osteopenia and OP patients and were associated with changes in BMD. Another study used circulating blood samples from post-menopausal women (divided into three groups: 24 normal, 30 osteopenia, and 24 OP) proved that the up-regulation of miR-194-5p in OP patients could serve as a biomarker of PMO (Meng et al., 2015). In addition, serum differentially expressed miR-140-3p and miR-23b-3p from patients with OP were validated in three groups (28 osteopenia, 26 OP and 21 osteoporotic hip fracture). The results showed that miR-140-3p and miR-23b-3p may be biomarkers of PMO (Ramirez-Salazar et al., 2018). Panach et al. (2015) analyzed serum samples from 15 patients with osteoporotic fracture and 12 controls by RT-PCR and identified three up-regulated miRNAs in osteoporotic fracture patients, i.e., miR-122-5p, miR-125b-5p, and miR-21-5p. Bedene et al. (2016) studied the plasma samples of 74 post-menopausal women and analyzed the expression and function of miRNAs from 17 PMO patients and 57 normal controls by qRT-PCR. They found that miR-148a-3p was a potential biomarker for PMO. Meanwhile, miR-122-5p and miR-21-5p can be used as biomarkers for the diagnosis of OP in other reports. Liu Y. et al. (2015) demonstrated that miR-331 (also known as: miR-331-3p) is a potential biomarker of OP by analyzing the differentially expressed miRNAs in bone tissues of 27 osteoporotic patients and 39 healthy controls in Array Express database.

### Systems-Based Pathogenesis Investigation

#### Tools and Analytical Methods

To decode the pathogenic role of the collected miRNAs in OP, we performed functional analysis from an integrated “miRNA-gene-pathway” angle using computational tools and methods. Among them, Gene ontology (GO) involves gene and gene product vocabulary which can be divided into three categories: biological process (BP) and cell components (CC), molecular function (MF). The Kyoto Encyclopedia of Genes and Genomes (KEGG) is a manually created database, where the biochemical processes of cells, such as membrane transport, cell cycle, metabolism and signal transmission, are illustrated. Ingenuity Pathway Analysis (Kramer et al., 2014) (IPA) contains data from international journals and relevant well-known databases. It provides functional choices for gene enrichment, including species, tissues, diseases, data credibility and mutations. The Database for Annotation, Visualization and Integrated Discovery (DAVID, version 6.7) (Huang da et al., 2009) is a free and online tool for comprehensive analysis of the large-scale gene or protein functions.

#### Gene-Level Analysis

To investigate the regulatory role of collected miRNAs in OP pathogenesis, the target genes of the OP miRNAs were identified by integrating the miRNA–gene relationships from different databases (Lin et al., 2018). Then, a total of 279 genes associated with OP occurrence and development were manually collected and mapped by mining previously published citations in PubMed (see Supplementary Table A1) to decipher the functional role of miRNAs in regulating OP-associated genes and pathways, which would help the understanding of OP pathogenesis and therapeutics.

Gene ontology analysis was performed on the identified targets of the collected OP miRNAs using the online tool DAVID (Huang da et al., 2009). Here the false discovery rate was applied to adjust raw *p*-values and significant terms (adjusted *p*-value < 0.05) at BP, CC, and MF level, summarized in Supplementary Table A2. To understand the function of genes in OP pathogenesis, the 10 most significant terms at the BP, CC, and MF levels were selected for further analysis. As shown in Table 2, the terms significantly enriched in BP were “positive regulation of transcription from RNA polymerase II promoter,” “positive regulation of transcription, DNA-templated,” “negative regulation of transcription from RNA polymerase II promoter,” “protein phosphorylation,” “transcription from RNA polymerase II promoter,” and “negative regulation of transcription, DNA-templated.” Jin et al. (2009) reported that the increased transcription of the proven COLIA1 polymorphic variants of OP-related genes may lead to a reduction in BMD. Sun et al. (2017) found that the reduction of LRP5 and beta-catenin leads to reduced osteoblast activity. Here the enriched BP terms were closely related to changes in bone metabolism, which may contribute to OP progression. For example, the proliferation, differentiation, and apoptosis process in osteoblasts and osteoclasts, as well as in their precursor cell lines, may affect the metabolic balance of bone formation and bone resorption. The terms significantly enriched in the CC domain included “nucleoplasm,” “cytosol,” “cytoplasm,” “nucleus,” “membrane,” “extracellular exosome,” “cell–cell adherens junction,” and “focal adhesion.” The changes in these key cell components were crucial to OP development, and changes in bone mass were ultimately affected by the composition and structure of the bone cells. The terms significantly enriched at MF level were “protein binding,” “transcription factor binding,” “chromatin binding,” “protein kinase binding,” “protein serine/threonine kinase activity,” “protein kinase activity,” “transcription factor activity, sequence-specific DNA binding,” and “cadherin binding involved in cell–cell adhesion.” These terms were also found used in literature on the occurrence and development of OP. For example, RUNX2 has been reported to stimulate osteoblast differentiation (Byun et al., 2014). AMP-activated protein kinase (AMPK) affects bone loss, increasing the risk of osteoporotic fracture (Jeyabalan et al., 2012). Based on the GO analysis, the molecular pathogenesis of OP could be well-explained, which is propitious to the precision diagnosis, treatment, and prognosis of OP.

**TABLE 2:** The 10 gene ontology terms most significantly enriched by targets of the reported miRNAs.

Category	GO terms	Number of enriched genes	Adj. *p*-value
BP	Positive regulation of transcription from RNA polymerase II promoter	281	1.37E-23
	Positive regulation of transcription, DNA-templated	246	2.14E-22
	Negative regulation of transcription from RNA polymerase II promoter	247	4.10E-22
	Protein phosphorylation	223	1.72E-21
	Transcription from RNA polymerase II promoter	232	5.30E-21
	Negative regulation of transcription, DNA-templated	234	8.09E-21
	Positive regulation of apoptotic process	185	1.38E-20
	Ephrin receptor signaling pathway	223	1.69E-20
	Viral process	214	6.61E-20
	Cell–cell adhesion	260	1.18E-19
CC	Nucleoplasm	406	4.14E-29
	Cytosol	463	8.54E-26
	Cytoplasm	477	2.18E-25
	Nucleus	468	7.60E-25
	Membrane	265	2.38E-22
	Extracellular exosome	346	5.28E-18
	Cell–cell adherens junction	219	5.42E-14
	Focal adhesion	562	1.03E-11
	Intracellular membrane-bounded organelle	562	1.03E-11
	Protein complex	163	1.87E-11
MF	Protein binding	180	3.16E-18
	Transcription factor binding	400	5.37E-16
	Chromatin binding	188	1.67E-12
	Protein kinase binding	141	8.16E-11
	Protein serine/threonine kinase activity	163	1.06E-10
	Protein kinase activity	112	1.39E-10
	Transcription factor activity, sequence-specific DNA binding	133	1.12E-09
	Cadherin binding involved in cell–cell adhesion	70	6.65E-08
	Ubiquitin protein ligase binding	62	6.74E-08
	Enzyme binding	115	2.44E-07

*Adj.*p*-value, adjusted *p*-value; BP, biological process; CC, cellular component; GO, gene ontology; MF, molecular function.*

The identified miRNA targets were further mapped and compared with those of collected OP-associated genes. A final total of 96 genes were found to be overlapped and regulated by 19 OP miRNAs (see Supplementary Table A3). Among them, six miRNAs, i.e., miR-23b-3p, miR-21-5p, let-7c, miR-148a-3p, miR-124-3p, and miR-503-5p, were extracted for regulatory mechanism analysis, as the targets of these miRNAs were closely involved in OP-associated processes such as estrogen–endocrine, WNT/β-catenin, and RANKL/RANK/OPG signaling. As shown in Supplementary Figure A1, most of these genes were independently regulated by single miRNAs in the network whereas some key players, including VEGFR, SP1, CDC25A, CDKN1A, were co-regulated by multiple miRNAs.

#### Pathway-Level Analysis

KEGG (Kanehisa and Goto, 2000) database and IPA (Kramer et al., 2014) were used to analyze the identified targets of OP miRNAs using the DAVID and IPA tools, respectively. The raw *p*-values were adjusted using the false discovery rate and enriched terms with statistical significance (adjusted *p*-value < 0.05) are summarized in Supplementary Table A4.

The 10 most significantly enriched KEGG and IPA signaling pathways were selected for further analysis. In KEGG, as shown in Table 3, some of these pathways were specific to cancer development, such as pancreatic cancer and prostate cancer. Accumulated evidence has proved that bone metastasis and bone loss are significant symptoms in these cancers, especially in the later stages (Chih et al., 2016; Poulsen et al., 2019). According to previous reports, p53 signaling pathway is involved in regulating osteoblast production and apoptosis and affecting BMD (Zhen et al., 2014; Liu W. et al., 2015). In addition, other items with significant enrichment have been reported to be involved in the occurrence and development of OP. For example, serum transforming growth factor β1 (TGF-β1) has been shown to inhibit the expression of sclerostin levels, which is negatively regulated by WNT signaling pathway, thus mediating bone turnover (Cheng et al., 2016). The TGF-β/smad2/COL4A1 pathway in TGF-β signaling could enhance the ability of BMSCs to differentiate from adipocytes, and weaken the differentiation of osteoblasts, therefore affecting bone metabolism and bone mass (Qiu et al., 2018). The MAPK signaling pathway plays a key role in the formation of osteoclasts, and selective MAPK inhibitors have a significant effect in PMO treatment (Li et al., 2016). The 10 most significantly enriched IPA pathways included: “Molecular Mechanisms of Cancer,” “p53 Signaling,” ^“^HGF Signaling,” “Glucocorticoid Receptor Signaling,” “Pancreatic Adenocarcinoma Signaling,” “ERK/MAPK Signaling,” etc. Previous studies found that some of signaling pathways involved in the bone cell differentiation and apoptosis processes that affect bone metabolism and BMD. Such as “p53 Signaling” (Zhen et al., 2014; Liu W. et al., 2015), “AMPK Signaling” (Li et al., 2011), “RANK Signaling in Osteoclasts”(Yu et al., 2016), “Wnt/β-catenin Signaling” (Rossini et al., 2013), “Estrogen Receptor Signaling” (Amorim et al., 2018), etc. Moutsatsou et al. (2012) reviewed the adverse effects of glucocorticoid receptor signaling pathway on osteocytes leading to OP. Kim et al. (2013) demonstrated that activation of MAPK/ERK1/2 signaling pathway could promote incremental expression of osteoblasts through *in vitro* animal cell experiments. Xi et al. (2015) demonstrated that PI3K/AKT Signaling is involved in many processes of osteoblast metabolism, thus affecting bone formation. In particular, the target genes of these biomarkers are enriched in signaling pathways, and many related reports are involved in the metabolic process of OP. Especially, the target genes related to OP are almost enriched in the center of these important pathways. These genes are closely regulated by miRNA biomarkers, which further proves the reliability of our research and analysis.

**TABLE 3:** The 10 pathways most significantly enriched by targets of the reported miRNAs.

Category	Pathway terms	Number of enriched genes	Adj. *p*-value
KEGG	Pathways in cancer	140	1.29E-17
	Proteoglycans in cancer	50	3.32E-11
	Prostate cancer	51	1.40E-10
	Renal cell carcinoma	42	3.74E-09
	Small cell lung cancer	42	1.33E-08
	FoxO signaling pathway	42	7.83E-08
	Pancreatic cancer	33	6.32E-07
	Hepatitis B	55	5.36E-06
	Chronic myeloid leukemia	27	7.45E-06
	Non-small cell lung cancer	36	1.67E-05
IPA	Molecular mechanisms of cancer	151	7.26E-28
	Senescence pathway	61	1.15E-19
	HGF signaling	63	6.08E-19
	p53 signaling	61	1.44E-17
	Hepatic fibrosis signaling pathway	71	1.45E-17
	Role of tissue factor in cancer	118	3.04E-17
	NGF signaling	83	6.55E-17
	ERK/MAPK signaling	62	7.21E-17
	Glucocorticoid receptor signaling	60	3.21E-16
	Pancreatic adenocarcinoma signaling	74	1.45E-15

*Adj.*p*-value, adjusted *p*-value; IPA, ingenuity pathway analysis; KEGG, kyoto encyclopedia of genes and genomes.*

Considering the complexity of OP, an integrated “miRNA–gene–pathway” analysis was conducted for the systems-level decoding of OP pathogenesis. As a biomarker for PMO diagnosis, miR-133a-3p regulates important target genes related to OP, such as VEGFA, SP1, COL1A1, EGFR, etc. These genes are concentrated in key sites of pathways closely related to OP, such as “Osteoarthritis Pathway,” “MAPK signaling pathway,” “TGF-beta signaling pathway,” “Glucocorticoid Receptor Signaling,” “ERK/MAPK Signaling,” etc. In particular, target gene SP1 plays an important role in signal transduction in these pathways. MiR-142-3p, a potential biomarker of PMO, and its target genes (CCND1, STAT1, ACVR1, TNFRSF11B, IRS2, CDC25B) also enrich and participate in signal transduction in these important pathways. In addition, CCND1, ACVR1 are enriched in Wnt/beta-catenin Signaling and IRS2 in RANK Signaling in osteoclasts. These signaling pathways have been previously reported to be closely related to bone metabolism in OP, while other target genes of miR-194-5p (target OP-associated genes: ACVR2B, PRKAR1A, RB1, SERPINE2, etc.), miR-21-5p (target OP-associated genes: TGFB1, JAG1, THBS1, SP1TGFB1, TNSF11B, EGFR, etc.), miR-23b-3p (target OP-associated genes: CCND1, FGF2, PDGFA, LRP5, etc.), and miR-30b-5p (target OP-associated genes: CCNE2, ACVR1, SMAD1, etc.), which are potential biomarkers of PMO and also mostly concentrated in the key sites of these pathways. Here miR-21-5p was found to be down-regulated in the serum of patients with OP and so could be used as a diagnostic biomarker for PMO. Interestingly, its target gene, TGFB1, is involved in extracellular signal transduction through the MAPK signaling pathways; thus miR-21-5p may affect the metabolism of bone cells by regulating TGFB1 in this pathway, influencing the occurrence and transformation of OP. In addition, the target genes (CCND1, CCNE2, CDKN1A, VEGFA, FGF2, CHEK1, etc.) of miR-503-5p, which are potential therapeutic target for the treatment of PMO, are also enriched in some important pathways, such as “Cell cycle,” “Pathways in cancer,” “p53 signaling pathway,” “AMPK signaling,” “PI3K/AKT signaling,” “Wnt/beta-catenin Signaling,” et al. Another miRNA, Seeliger et al. (2014) and Bedene et al. (2016) showed that miR-148a-3p was a potential biomarker for the diagnosis and treatment of PMO. Its target genes ACVR1, DKK1, and ACVR2B are all enriched in the Wnt/beta-catenin Signaling pathway. Therefore, miR-148a-3p may affect bone metabolism in patients with OP by regulating these key factors through the Wnt/beta-catenin signaling pathway. Furthermore, pathways enriched by miRNAs such as miR-93-5p (target OP-associated genes: CDKN1A, PIK3R1, TNFSF11, PPARG, BMP2, etc.), miR-124-3p (target OP-associated genes: JAG1, SP1, NR3C1, etc.), miR-125b-5p (target OP-associated genes: FGFR2, VDR, ETS1, CDKN2A, etc.), miR-23a-3p (target OP-associated genes: PDGFA, LRP5, PTPN11, etc.), miR-24-3p (target OP-associated genes: PCNA, TGFB1, CDKN2A, CCNA2, etc.), miR-25-3p (target OP-associated genes: IRS2, SOX4, SP1, etc.), and miR-181c-5p (target OP-associated genes: ITGB8, HLA-B, etc.) are also reported to be involved in OP. For example, considering the regulation between OP miRNAs and their targets BMP2 and SP1 in the ‘Osteoarthritis Pathway” and “TGF-beta signaling pathway,” BMP2 are functional in osteoblast differentiation (Yoo et al., 2017). According to the identified miRNA–gene relationships, BMP2 are the direct targets of miR-93-5p, and respectively SP1 is co-regulated by miR-93-5p, miR-124-3p, miR-133a-3p, miR-21-5p, and miR-25-3p. In particular, LRP5/6 mediates signal transduction on the cell membrane of osteoblasts, regulating the expression of osteoblasts by interacting with extracellular WNT and DKK1 (Iozzi et al., 2012). Meanwhile, miR-21-5p, miR-23a-3p, and miR-23b-3p has been reported as a potential biomarker in OP diagnosis, with its target gene LRP5/6 acting as a transmembrane protein transduction signal in the WNT signaling pathway. In addition, plenty of studies have shown that miR-125b is up-regulated in blood and bone tissue in OP (Chen S. et al., 2014; Seeliger et al., 2014), and its target gene, FGFR2, is involved in the MAPK signaling pathway, RANK signaling in osteoclasts, and p53 signaling; thus miR-125b-5p may regulate these pathways by targeting FGFR2. All these genes and pathways have been reported to be associated with OP development. Therefore, these target genes and related signaling pathways are regulated by these miRNA biomarkers, which provide new insights in systems level OP pathogenesis decoding.

**FIGURE 3:**
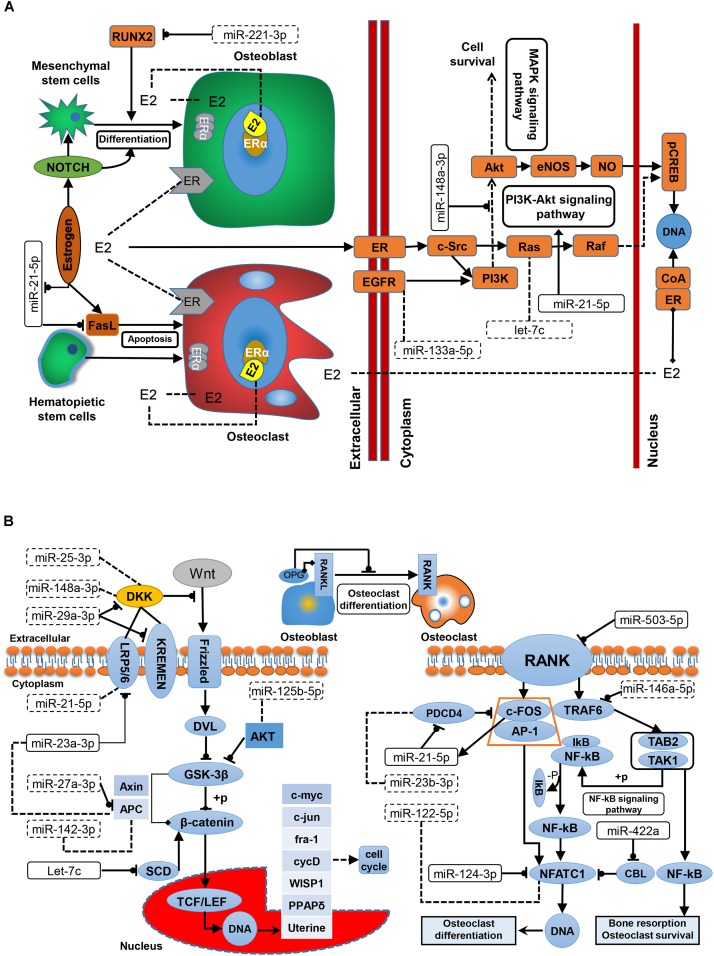
The regulatory role of miRNAs in OP-associated pathways. **(A)** In the estrogen signaling pathway. Estrogen-mediated systemic signaling has protective effects on BMD in OP. **(B)** In the WNT/β-catenin and RANKL/RANK/OPG pathway. The WNT/β-catenin signaling pathway is the major signal transduction pathway regulating osteoblast differentiation. The RANKL/RANK/OPG pathway may regulate the development of osteoblasts and osteoclasts. E2, 17β-estradiol; EGFR, epidermal growth factor receptor; ER, estrogen receptor; LRP, low-density-lipoprotein receptor-related protein; OPG, osteoprotegerin; RANKL, receptor activator for nuclear factor-κB ligand; RANK, receptor activator for nuclear factor-κB; RUNX2, Runt-related transcription factor 2.

Since the development of OP is closely related to the dysfunction of three well-studied pathways, i.e., the estrogen–endocrine, WNT/β-catenin signaling, and RANKL/RANK/OPG pathways, exploring the underlying relationships between the reported miRNAs and these three pathways based on a systems biology viewpoint would enhance our pathogenic understanding of OP. Estrogen deficiency is the main cause of PMO, and estrogen replacement therapy is clinically effective for PMO. The relationship between estrogen signaling and OP miRNAs is shown in Figure 3A. Estrogen promotes the differentiation and activity of osteoblasts at the cellular level and inhibits osteoclasts. Fan et al. (2014) demonstrated that estrogen promotes the proliferation and differentiation of hBMSCs through Notch signaling in PMO patients, while Notch signaling promotes osteoblast differentiation and increases bone mass. Zhang et al. (2017) found that miR-221 inhibits the differentiation of osteoblasts by regulating RUNX2; therefore, miR-221 might be a potential target for OP treatment. The mechanism of Fas ligand (FasL)-mediated apoptosis has been widely studied; however, exactly how estrogen regulates the apoptosis of osteoclasts by targeting FasL remains controversial, and the source of FasL secretion is still unclear. Shao et al. (2015) showed that estrogen indirectly increases paracrine FasL in MSCs to promote osteoclast apoptosis and maintain bone mass. Wang et al. (2015) demonstrated that estrogen acts on osteoclasts through FasL secreted by osteoblasts to increase the apoptosis of osteoclasts. In addition, ERα induces the expression of FasL in osteoclasts and induces osteoclast apoptosis. Sugatani and Hruska found that estrogen reduced the effect of miR-21 on FasL autocrine and promoted the apoptosis of osteoclasts by down-regulating the expression of miR-21 (Sugatani and Hruska, 2013). It is believed that estrogen is ineffective in regulating FasL-mediated apoptosis in osteoblasts. Estrogen is essential for the prevention of bone loss in patients with PMO. ER-mediated nuclear-initiated biological signal transduction, estrogen-mediated membrane-initiated signal transduction, non-ER-mediated signal transduction, and ER ligand-independent signal transduction are the main estrogen signaling mechanisms (Cui et al., 2013). Current studies on the estrogen signaling mechanisms in bone tissue and cells have focused on ERα- and ER-ligand-independent pathways. Xiao et al. (2018) demonstrated in ovariectomized rat models that the over-expression of miR-148a in osteoporotic patients could inhibit the expression of phosphoinositol-3-kinase regulatory subunit 1 (PI3K) and phosphorylated protein kinase B (AKT) in osteoblasts *in vitro*, thereby inhibiting the growth of osteoblasts and promoting fineness. In addition, miR-148a-3p inhibited the expression of ERα protein. Another experimental study demonstrated that miR-21-5p can promote fracture healing by activating the PI3K/Akt signaling pathway (Liu et al., 2019). As illustrated in Figure 3A, Ras is involved in the ER-mediated membrane activation signaling pathway, and it is a target of let-7c. Thus let-7c may be involved in estrogen signaling, ultimately affecting bone cell metabolism.

According to previous reports, WNT signaling and the RANKL/RANK/OPG pathway may influence bone metabolism and bone mass. As described in Figure 3B, the classic WNT signaling pathway controls the metabolism of osteoblasts through WNT/β-catenin signaling. For example, miR-29a-3p reportedly down-regulates the antagonists DKK1 and Krenem2 to activate the WNT signaling pathway, therefore, may be helpful in the and treatment of PMO. Another two miRNAs, miR-25-3p and miR-148a-3p are involved in OP metabolism by targeting the WNT signaling pathway through OP-related genes. Dong et al. (2017) demonstrated that miR-23a-3p can regulate the expression of LRP5 through animal model experiments. Inhibiting the activity of miR-23a-3p can increase the expression level of LRP5 and thus increase the differentiation of osteoblasts. As a target of miR-27a, APC is inhibited, and miR-27a increases β-catenin to activate the WNT signaling pathway (Guo et al., 2018). Other studies have shown that miR-142-3p regulates APC activation of Wnt/β-catenin Signaling, while Hu et al. (2016) have demonstrated that miR-142-3p can directly inhibit the activity of β-catenin in Wnt/β-catenin Signaling. In addition, let-7c can inhibit Wnt/beta-catenin signaling by reducing the expression of stearoyl-CoA desaturase 1 (SCD1) in OP patients, thus reducing osteoblast differentiation (Zhou et al., 2019). AKT both promotes and inhibits GSK-3β phosphorylation. miR-125b is a biomarker in OP treatment, and studies have shown that its down-regulation inhibits PI3/AKT/GSK-3β and WNT/β-catenin signaling in non-small cell lung cancer (Wang et al., 2017). Despite a current lack of evidence, this mechanism may potentially operate in bone tissue. In the OPG/RANKL/RANK pathway, osteoblasts interact with osteoclasts. Competitive binding of OPG secreted by osteoblasts with RANKL inhibits bone resorption and induces the apoptosis of osteoclasts, mediated by the RANKL/RANK signaling pathway. Activation of the RANKL/RANK signaling pathway promotes the proliferation and differentiation of osteoclasts, and is the key mechanism of osteoclast regulation. A receptor of RANK on the surface of osteoclasts binds to RANKL to recruit TNF receptor activator 6 (TRAF6) and binds to form a trimer in cells. Nuclear factor-κB (NF-κB) is then activated and a series of processes, including regulation of c-FOS (an important transcription factor related to osteoclast development) and reactive protein 1 (AP-1), leading to the formation of a trimer activating T-cell nuclear factor protein 1 (NFATC1) expression and regulating the transcriptional expression of related genes (Chen C. et al., 2014). Chen C. et al. (2014) demonstrated in ovariectomized rats that over-expression of miR-503 directly inhibited the transduction of RANKL/RANK signals into cells in PMO, information that provided a new strategy for OP treatment. miR-21-5p is a biomarker in the diagnosis of PMO (Yavropoulou et al., 2017), and RANKL/RANK in osteoclasts may bind to the promoter of miR-21-5p by stimulating c-FOS, as well as PDCD4, which inhibits c-FOS and is inhibited by the regulation of miR-21-5p, thus forming a positive feedback loop in osteoclasts. Over-expression of miR-21-5p promotes osteoclast expression. Estrogen-induced ERα down-regulates miR-21-5p in this process, and indirectly increases the expression of FasL protein to promote osteoclast apoptosis. miR-146a-5p inhibits the differentiation and function of osteoclasts by regulating TRAF6. NFATC1, as a key transcription factor in the RANKL/RANK pathway, is down-regulated by CBL degradation, inhibiting the early formation of osteoclasts (Kim et al., 2010). As a potential PMO biomarker, miR-422a has been reported to be up-regulated in the monocytes of PMO patients, and negatively correlated with CBL expression. Lee et al. (2013) demonstrated that miR-124 regulated osteoclast production by decreasing NFATC1 expression and inhibiting the activity of osteoclast precursors.

#### Cross-Level Analysis

The development of OP is affected by a combination of genetic and non-genetic factors, thus cross-level analysis of OP pathogenesis, i.e., the integration of molecular events (e.g., miRNAs, genes, and pathways) with signatures at individual (e.g., clinical phenotype) and population level (e.g., lifestyle, living environment) is essential. The abnormal expression of miRNAs in OP patients regulates the function of their target genes and participates in some key biological signaling pathways associated with bone metabolism in OP. For example, miR-124, is also down-regulated in the serum of OP patients, and it may weaken the inhibition of NFATC1 and promote bone resorption of osteoclasts. In addition, the over-expression of let-7c in Wnt signaling pathway reduces osteoblasts proliferation by inhibiting target genes SCD1 under oxidative stress (Zhou et al., 2019). The interactions between miRNAs and target genes affect bone metabolism balance, and the complexity of miRNA–gene-pathway regulation may explain the heterogeneity of OP at the individual level.

In addition to molecular factors, surgical intervention, lifestyle, and living environment are important in OP initiation and progression. For example, PMO often develops following physiological menopause; however, ovariectomy may also lead to estrogen deficiency, another reason for the high incidence of OP in post-menopausal women. Previous studies have shown that appropriate physical exercise can increase BMD, and a healthy diet and adequate nutritional intake are also positive lifestyle changes that influence OP prevention and treatment. Al-Raddadi et al. (2018) investigated OP risk factors among Saudi Arabian female adolescents and found that insufficient vitamin D and calcium intake, inadequate dairy consumption, lack of exercise, and insufficient sunlight exposure were associated with OP. A high-sugar diet has a negative impact on bone development, and obesity caused by a high-fat diet is harmful to bone metabolism. Adipocytes are derived from BMSCs, and miR-221 may inhibit RUNX2-induced differentiation of bone marrow MSCs into osteoblasts, resulting in increased adipocyte differentiation. Tian et al. (2017) conducted a cross-sectional study of PMO and SOP in Gansu Province in China. They found that PMO in women was closely related to age at menopause, duration of post-menopause, body mass index (BMI), alcohol consumption, and educational level, whereas SOP in men was significantly correlated with age, BMI, smoking habits, alcohol consumption, physical exercise, and sun exposure. We found that miR-21-5p affects the expression of osteoclasts by regulating key factors in the RANK signaling pathway. Here we consider that changes in lifestyle and diet may affect the expression of miRNAs and some key target genes and affect individual clinical phenotypes. Previous studies have reported that miR-122a and miR-125b are down-regulated in mice with chronic vitamin E deficiency (Gaedicke et al., 2008). Furthermore, smoking and drinking can also lead to changes in the concentration of miR-122a and miR-148a *in vivo* (Seike et al., 2009; Stanitz et al., 2015), and the influence of these exposure risk factors can activate the occurrence of OP-related mechanisms. Therefore, an understanding of the functional role of non-genetic factors, especially lifestyle and environmental regulators, is beneficial to the early prevention and treatment of OP.

Osteoporosis prevention and treatment remains a challenge and the development of integrative methods for precision medicine in the management of OP is urgently required. On one hand, miRNAs hold the power to enable the decoding of the hidden pathogenesis of OP and are potential drug targets for OP therapy. On the other hand, non-drug therapies, i.e., lifestyle and environmental interventions, are also effective in OP management and would be advantageous in personalized OP treatment and healthcare.

## Discussion

Osteoporosis is a systemic bone disease with increased incidence with age. Due to their pathological role in the development of OP, miRNAs have gradually been validated as biomarkers for OP monitoring, such as early diagnosis, prognosis tracking, and personalized therapy.

In this article, a total of 28 previously reported OP miRNAs were collated and analyzed, based on systems biology approaches. Most of these miRNAs were of value in OP diagnosis, therapeutics and prognosis. To decode the regulatory role of the collected miRNAs, the targets were then identified from public databases. OP-associated genes were mined and integrated for a systems-level analysis of OP pathogenesis. Based on functional enrichment analysis, significantly enriched KEGG and IPA terms were discovered, such as “ERK/MAPK signaling pathway,” “Glucocorticoid Receptor Signaling,” “TGF-β signaling pathway,” “p53 signaling,” “Estrogen Receptor Signaling,” “RANK Signaling in Osteoclasts,” and “Wnt/β-catenin Signaling.” All of these pathways have been reported to be associated with bone metabolism in OP. Moreover, most of the target genes of biomarker miRNAs were located at the key sites of these pathways, which convinced us of the functional importance of miRNAs in OP development. For example, miR-21 may affect the metabolism of osteoporotic osteocytes by regulating TGF-β1 in the FoxO and MAPK signaling pathways, and miR-503 regulates RANK, participating in osteoclast proliferation, differentiation, and activity. According to previous reports, the development of OP is closely related to dysfunction in three well-known pathways, i.e., the estrogen–endocrine pathway, the WNT/β-catenin signaling pathway, and the RANKL/RANK/OPG pathway. To better understand the heterogenicity of OP, the relationships among miRNAs, pathways, and different OP subtypes were closely analyzed. For example, miR-221 is a potential treatment of PMO. Due to estrogen deficiency, miR-221 inhibits RUNX2 to regulate MSC differentiation into osteoblasts. MiR-27a and miR-29a participate in WNT/β-catenin signaling by regulating the associated target genes. But these miRNAs as biomarkers of OP need to be further validated. miR-21 and miR-124a regulate gene PDCD4 and NFATC1, respectively, and have opposite effects in the RANKL/RANK signaling pathway. In addition to genetic factors, some non-genetic elements, e.g., lifestyle and living environment, were found to be essential in OP epidemiology; thus cross-level analysis is of great significance for OP precision medicine and healthcare.

This study provides a comprehensive review of reported OP miRNAs and integrates “miRNA–gene–pathway” knowledge to explain the pathogenic role of miRNAs in OP genesis; however, some limitations exist. First, the alteration of the collected miRNAs is only validated in the experimental stage, e.g., using cell lines, model animals, and human samples; translational application of biomarkers in OP clinical management needs to be promoted. Second, the effects of non-genetic factors on miRNA–OP interactions are insufficiently investigated. With such complexity and heterogenicity in OP development, the importance of lifestyle and environmental factors on OP epigenetics should be reasonably considered. Third, the initial intention was to consider the interaction mechanism between different types of OP and miRNA alterations, but due to the limitations of existing research data, it has not been better expanded. Finally, but importantly, only 28 miRNAs were recorded in this study. Considering the papers for miRNA selection, limitations still need to be concerned. For example, many of the studies identified the change of miRNA expression based on small sample validation, further clinical tests using multi-center sample sources should be performed to evaluate the sensitivity and specificity of the miRNA candidates. On the other hand, it is difficult to verify whether a miRNA could serve as a true biomarker due to the design of research scheme, the selection of reference standard, and the reliability of result evidence. In this review, we mainly selected miRNAs validated by low-throughput experiments such as PCR to ensure the biomarker potential. For clinical translation, integrated computational prediction and experimental validation should be encouraged for decoding miRNAs in OP pathogenesis at the systems biology level. With advances in experimental and computational biology, more miRNA biomarkers will be identified and verified for the personalized prevention and treatment of OP, and methodology used in OP data analysis can reasonably be expected to improve, creating a systematic framework for use in intelligent medicine and healthcare.

## Data Availability Statement

The data supporting this review are from previously reported studies and datasets, which have been cited. The processed data are available at tables/figures in the Supplementary Material from the corresponding author upon request.

## Author Contributions

HH, XH, and YL collected the data. HH, XH, YZ, RW, and YL reviewed the data and performed the bioinformatics analyses. All authors drafted and revised the manuscript. HH and XH contributed equally to this study. BS and YL conceived and supervised this study jointly.

## Conflict of Interest

The authors declare that the research was conducted in the absence of any commercial or financial relationships that could be construed as a potential conflict of interest.
