# Supplementary material for: MicroRNA Alterations for Diagnosis, Prognosis, and Treatment of Osteoporosis: A Comprehensive Review and Computational Functional Survey
Source: Front Genet. 2020 Mar 3;11:181. doi: 10.3389/fgene.2020.00181 (PMC7063117; doi:10.3389/fgene.2020.00181)
Supplement: Supplementary file 1 [file Image_1.pdf]

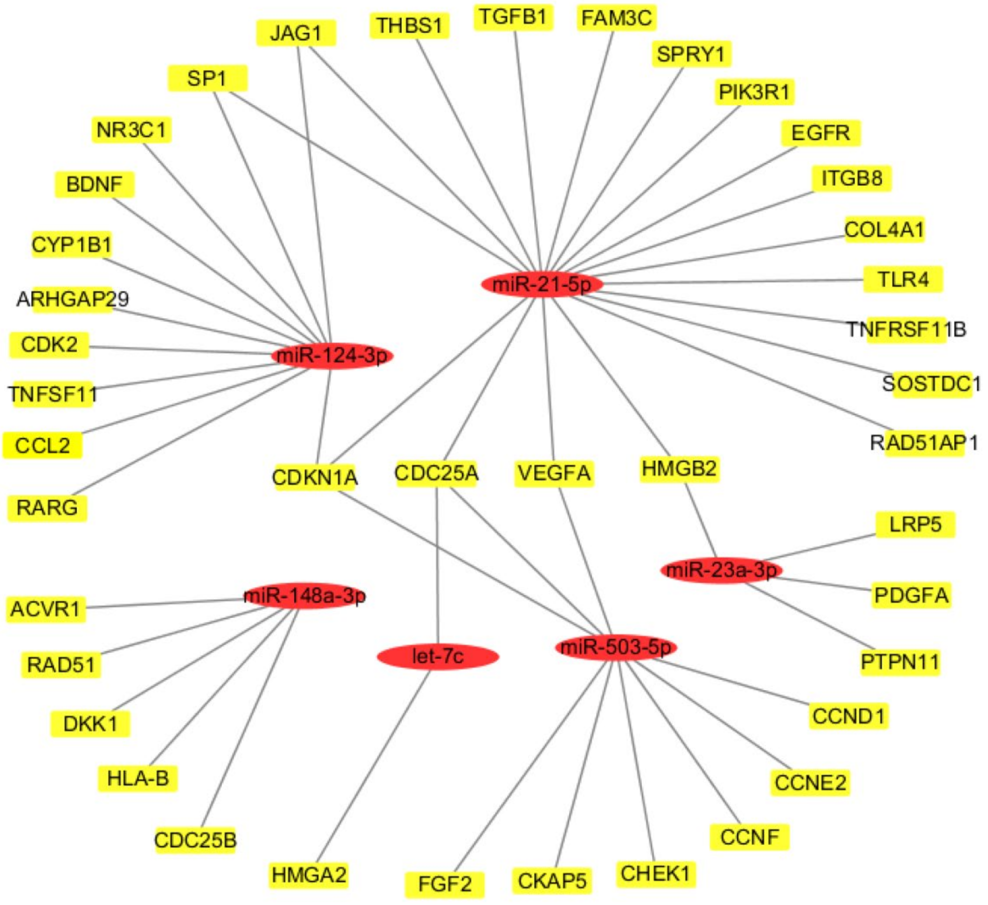

Figure A1. Six miRNAs and their targeted OP-associated genes. The selected six miRNAs are closely related to three well-studied OP pathways, i.e., estrogen–endocrine, WNT/β-catenin signaling, and RANKL/RANK/OPG. Red ellipses and yellow rectangles represent miRNAs and genes, respectively.
